# Cellulitis Occurring 21 Years After Radiofrequency Ablation for Breast Cancer: A Case Report

**DOI:** 10.7759/cureus.107071

**Published:** 2026-04-14

**Authors:** Tatsunori Higashi, Shoji Oura

**Affiliations:** 1 Department of Surgery, Kishiwada Tokushukai Hospital, Kishiwada, JPN

**Keywords:** breast cancer, cellulitis, dystrophic calcifications, late complications, radiofrequency ablation

## Abstract

A 67-year-old woman had undergone radiofrequency ablation (RFA) for right breast cancer at a university hospital 21 years before and visited our hospital for annual breast surveillance after finishing the 10-year follow-up. During the annual breast surveillance, unexplained and unintentional weight loss made the subcutaneous fat just above the RFA nodule thin. It caused cellulitis due to the RFA nodule-related stimuli to the overlying skin. Image evaluation clarified the strong adhesion between the RFA nodule and the overlying skin. After recovery from cellulitis, the patient requested resection of the RFA nodule to prevent recurrence. The patient, however, already had marked asymmetry of the breasts due to the radiation-induced shrinkage of the right breast. The patient, therefore, underwent breast surgeries as follows to get better cosmetic outcomes. The patient underwent RFA nodule resection via a retromammary space approach through a lateral vertical skin incision, achieving a difficult but successful complete removal of the RFA nodule. After that, the shrunken and hard right mammary gland was partially incised in several linear fashions from the retromammary space, followed by immediate breast augmentation using the latissimus dorsi musculocutaneous flap. Additionally, the left breast was treated with inverted T reduction mammoplasty, successfully leading to the favorable cosmesis of the breasts. The patient recovered uneventfully, is fully satisfied with the cosmetic outcomes of her breasts after the operation, and is scheduled to further receive annual breast surveillance on an outpatient basis. Breast surgeons should note that the RFA indication for breast cancer should not only include breast cancer characteristics but also late and aging-related complications.

## Introduction

Non-surgical ablation has long attracted physicians’ attention as a feasible alternative to surgical resection for various solid malignancies. Non-surgical ablation includes various modalities such as radiofrequency ablation (RFA), microwave ablation, cryoablation, and focused ultrasound ablation [1-5]. In particular, RFA and microwave ablation have already become standard therapies and have provided significant benefit worldwide for many patients with hepatocellular carcinoma.

Non-surgical ablation has also long been attracting the attention of breast surgeons as the ultimate breast-conserving therapy for breast cancer. Non-surgical ablation naturally does not require tissue resection, leading to excellent cosmesis. Surprisingly, RFA has recently been approved for insurance coverage in Japan, although certain prerequisites exist [6]. If it is proven that RFA can provide favorable clinical outcomes similar to breast-conserving therapy after long-term follow-ups in many breast cancer patients, local treatment for breast cancer could be markedly changed in the future.

Several studies have reported the favorable local control of RFA for breast cancer with limited follow-up periods up to 50 months. Although it is covered by insurance for breast cancer in Japan, Japanese guidelines currently recommend conducting it as a clinical trial [7]. Many medical facilities, therefore, are actually beginning to perform RFA for breast cancer in prospective clinical trials.

In addition to local control, breast surgeons should pay utmost attention to RFA-related complications when applying RFA to breast cancer [8]. RFA has possible thermal injuries to the overlying skin, local pain, and nipple retraction as acute-phase complications and absorption- and dystrophic calcification-related complications, i.e., skin retraction and oppression, as middle-phase complications [9]. However, little is known about the late-phase complications due to the small number of breast cancer patients who were treated by RFA and have survived without any recurrences for more than 10 years.

We herein report a patient who had undergone RFA for her breast cancer more than 20 years before, experienced unexplained and unintentional weight loss of 8 kg, and thereafter developed cellulitis near the RFA nodule due to the skin stimuli from the RFA nodule.

## Case presentation

**Figure 1:**
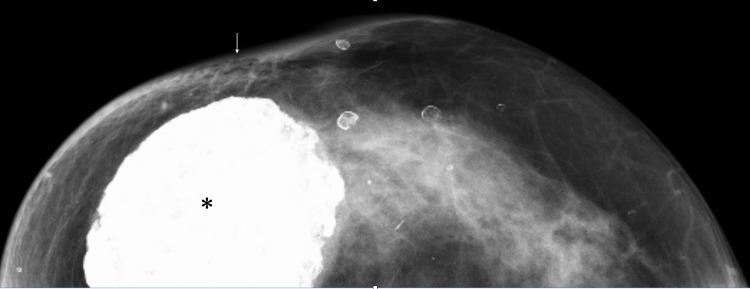
Mammography findings Mammography showed a very large dystrophic calcification (asterisk) and the slight skin retraction (arrow) at the skin just above the dystrophic calcifications.

A 67-year-old woman had undergone RFA and axillary dissection followed by adjuvant radiotherapy, i.e., unknown details of RFA procedures and adjuvant systemic therapies, for breast cancer at a university hospital 21 years before. She visited our hospital for annual breast surveillance after finishing the 10-year follow-up at the university hospital. Mammography 17 years after RFA showed a very large dystrophic calcification in the right breast and slight skin retraction just above the RFA-nodule (Figure 1).

**Figure 2:**
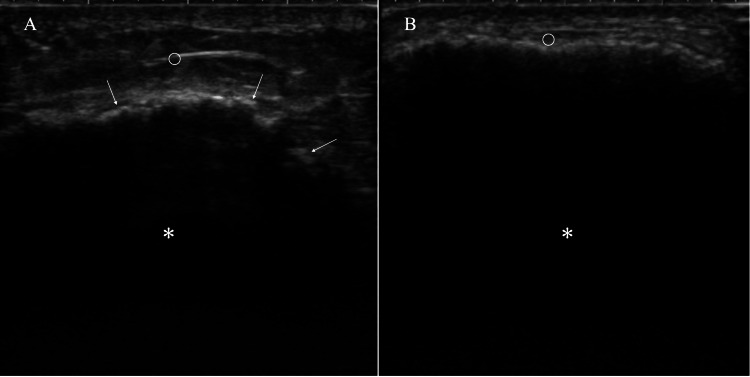
Ultrasound findings (A) Ultrasound showed subcutaneous fat approximately 1 cm thick (open circle) just above the RFA nodule (asterisk) despite the impossible observation of the internal structures of the RFA nodule due to the presence of dystrophic calcifications (arrows). (B) Ultrasound clarified the marked decrease of subcutaneous fat (open circle) just above the RFA nodule (asterisk) at the onset of cellulitis. RFA: radiofrequency ablation

Ultrasound showed no newly developed lesions in the breast, and an RFA nodule was encompassed by dystrophic calcifications, which made it impossible to evaluate the mass in detail due to strong ultrasound wave reflection (Figure 2).

**Figure 3:**
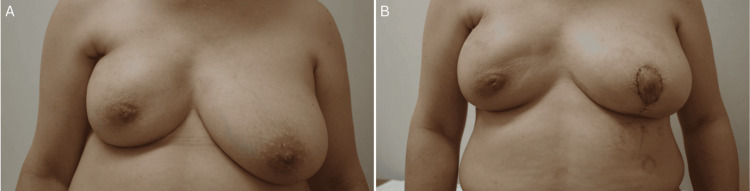
Local appearance of the breasts (A) Breasts after the resolution of cellulitis showed marked asymmetry due to the radiation-induced shrinkage of the right breast. (B) The patient got markedly improved cosmesis of the breasts just after the surgery and was fully satisfied with the cosmetic outcomes of her breasts.

The patient had markedly asymmetric breasts due presumably to the radiation-induced breast fibrosis and had unexplained and unintentional weight loss of 8 kg in the 20th year after RFA, leading to the decrease of subcutaneous fat volume just above the RFA nodule and the development of cellulitis around the RFA nodule (Figure 3A).

**Figure 4:**
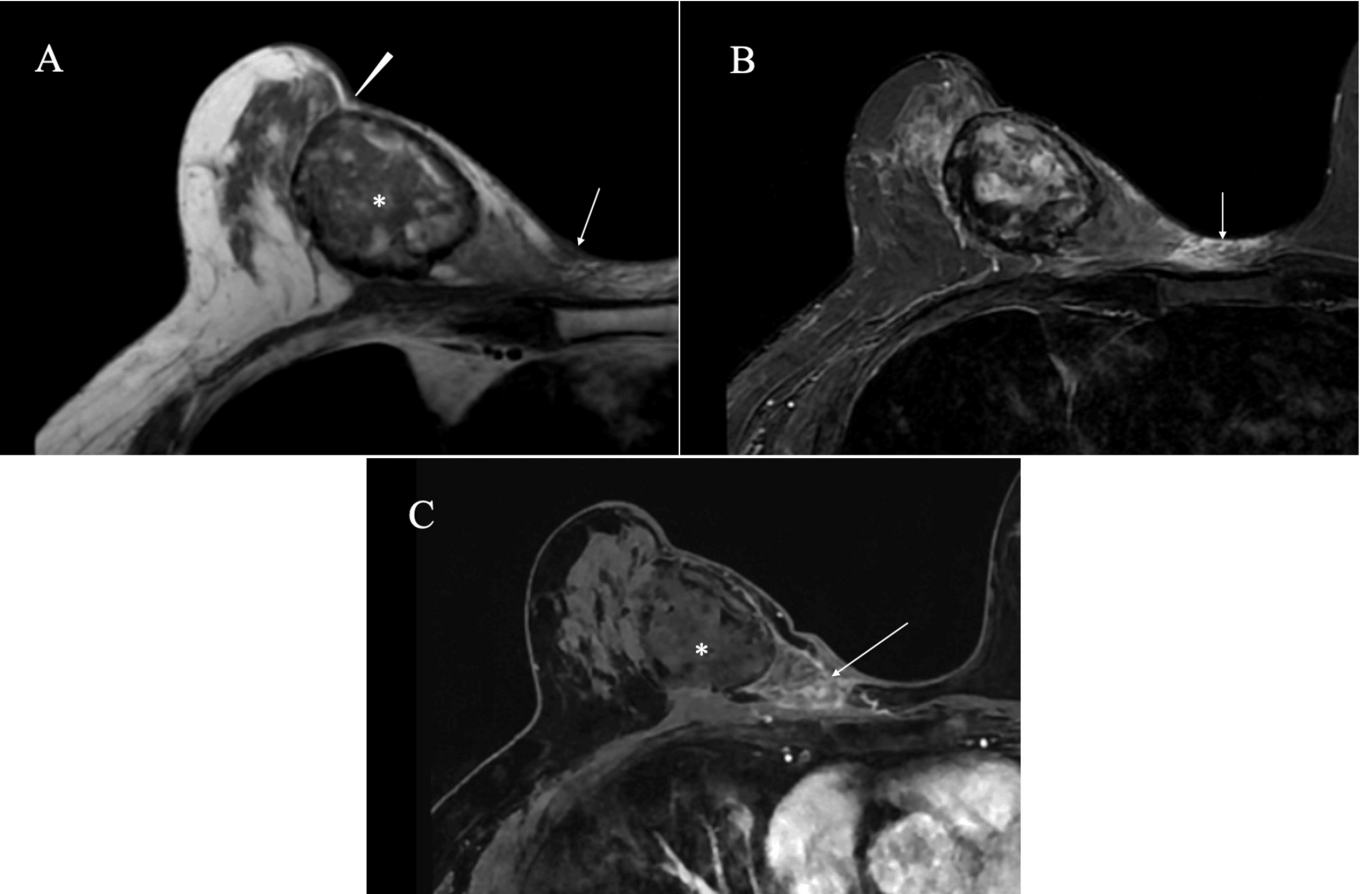
MRI findings (A) T1-weighted images showed an RFA nodule (asterisk), presumed adhesion between the RFA nodule and the overlying skin (arrowhead), and low signals at the skin (arrow) due to cellulitis. (B) MRI of the skin just above the sternum showed high signals (arrow) on T2-weighted images, suggesting inflammation, i.e., cellulitis. (C) Dynamic studies showed enhancement in the areas (arrow) medial to the RFA nodule, presumably due to cellulitis. MRI: magnetic resonance imaging, RFA: radiofrequency ablation

Magnetic resonance imaging (MRI) findings suggested an adhesion between the RFA nodule and the overlying skin, as well as skin inflammation and subcutaneous fat (Figure 4).

**Figure 5:**
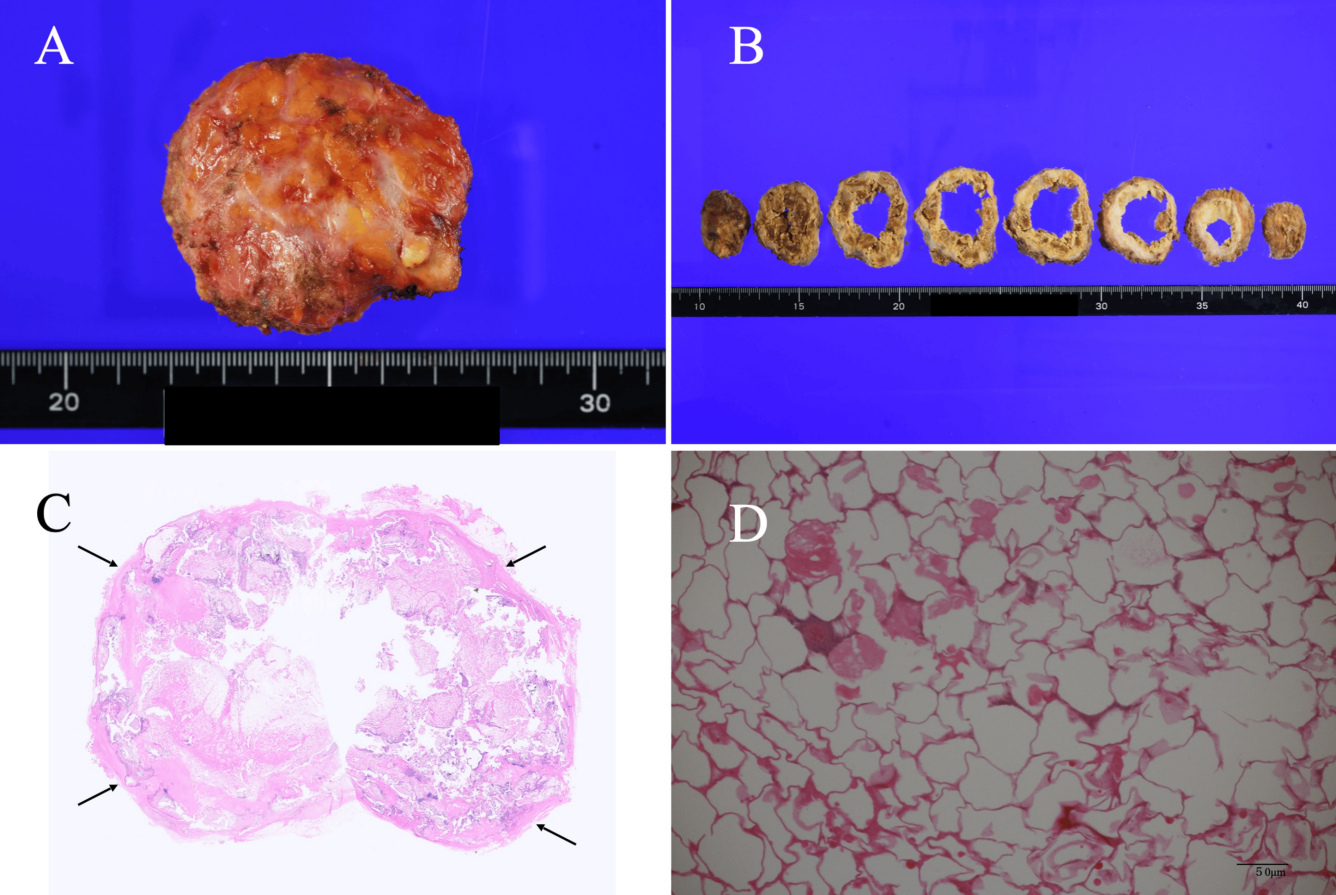
Pathological findings (A) The excised RFA nodule had distinct margins and an oval shape. (B) The cut surface lacked the vast majority of internal contents, presumably due to liquid, resulting in a pathological assessment limited to the rim areas. (C) The RFA nodule was encompassed by thick calcified tissues (arrows). (D) The magnified view showed marked fat necrosis in the areas immediately adjacent to the dystrophic calcifications. RFA: radiofrequency ablation

After the cellulitis resolved with conservative treatment, the patient strongly requested that we remove the RFA nodule to prevent recurrence of cellulitis. We judged that removal of the large calcified RFA nodule could prevent recurrence of cellulitis in the patient, but would further worsen her breast cosmesis. We therefore tried to improve the postoperative cosmetic outcomes not only by resecting the RFA nodule but also by performing immediate breast reconstruction on the right breast and reduction mammoplasty on the contralateral breast. We made a skin incision not on the overlying skin of the RFA nodule but on the lateral border skin of the right breast, followed by retromammary space dissection, and reached the RFA nodule base. We had difficulty dissecting the strong adhesion between the RFA nodule and the skin. Fortunately, we managed to completely enucleate the RFA nodule without skin damage, leaving no residual calcified RFA walls. The hard, shrunken mammary gland, caused by radiation-induced fibrosis, was successfully stretched by making several partial linear incisions in the mammary gland from the retromammary space, and was immediately augmented with the latissimus dorsi musculocutaneous flap. We thereafter performed a reduction mammoplasty to the left breast using inverted T procedures. Postoperative pathological study showed 60 mm of fat necrosis with scarring, calcifications, and ossifications (Figure 5).

The patient was unfortunately hospitalized for 16 days due to the continued high-volume lymph drainage, but recovered well without leaving any sequelae. These surgical procedures provided not supreme but favorable cosmesis, which was sufficient to satisfy the patient (Figure 3B). The patient is scheduled to continue receiving annual breast surveillance on an outpatient basis at our hospital.

## Discussion

Thermal injury is the most important side effect of RFA for breast cancer, especially in Japanese women who often have small breasts [9]. Various techniques have therefore been developed to prevent thermal injury during RFA, such as injecting a glucose solution into the space between the overlying skin and the breast cancer. Therefore, it is a prerequisite for breast surgeons to learn RFA techniques at a facility staffed by doctors skilled in RFA when obtaining insurance coverage for RFA in Japan. Additionally, it is reasonable that the insurance coverage defines the indication of RFA to be limited to breast cancer 1.5 cm or smaller in size, both for adequate ablation and for the avoidance of thermal injury [6].

Breast tissue naturally becomes necrotic after full thermal damage [10]. It is ideal for breast cancer patients treated with RFA to have no RFA nodules, as macrophages and other immune cells completely absorb the RFA-induced necrotic tissue. In the real RFA world, however, many patients have some kind of RFA nodules, like a boiled egg, consisting of necrotic breast tissue often surrounded by dystrophic calcifications. In fact, this patient had a very large calcified RFA nodule on mammography in the initial phase of annual follow-ups at our hospital.

Breast cancer patients can palpate the RFA nodules. It, therefore, is natural for some breast cancer patients treated by RFA to feel somewhat anxious about the RFA nodules. Thick subcutaneous fat tissue can give favorable cosmesis even to breast cancer patients with large calcified RFA nodules. In fact, this patient was extremely satisfied with the excellent cosmesis shortly after the RFA treatment despite the presence of a large RFA nodule up until the onset of breast shrinkage due to radiation-induced fibrosis [11]. On the other hand, this patient had recognized that her breasts had become gradually asymmetric due to the radiation-induced breast fibrosis. Breast specialists, therefore, should note that radiation-induced breast fibrosis can deteriorate breast cosmesis even when RFA can give excellent cosmesis to the patient.

Breast surgeons should further take the aging effect beyond 20 years on the breast treated by both RFA and radiotherapy into consideration to avoid cosmetic deterioration and late RFA-related events. Ultrasound clearly showed that unexplained and unintentional 8 kg weight loss had made the subcutaneous fat just above the RFA nodule thin in this case. It is well known that weight gain can be a risk factor for recurrence in luminal-type postmenopausal breast cancer patients [12]. Breast surgeons should also note that weight loss can lead to cellulitis around the large calcified RFA nodules.

Breast surgeons can most easily resect the RFA nodule through an anterior approach, especially when there is suspected adhesion with the overlying skin. An expert surgeon, having much surgical expertise for loco-regional recurrence of breast cancer, resected this RFA nodule, but needed a longer operation time than expected. Breast surgeons, therefore, should note that even severe adhesion between the RFA nodule and the overlying skin may be detached with cautious surgical procedures and should not hastily select an anterior approach for the resection of a calcified RFA nodule for better cosmetic outcomes.

It is a very important clinical matter for breast surgeons to prevent or minimize RFA-nodule formation. Vacuum-assisted biopsy can provide useful pathological findings, especially about the cell viability shortly after RFA, when using large-gauge needles. Multiple harvesting of trans-RFA nodules can lead to the formation of non-thermocoagulated tissue within the RFA nodules. It may accelerate the absorption of RFA induration due to infiltration of phagocytic immune cells, possibly preventing dystrophic calcification.

This is an extremely rare case of RFA nodule, which caused cellulitis around the adhesion between the RFA nodule and the overlying skin more than 20 years after the RFA treatment for breast cancer. RFA can be a promising local therapy for breast cancer and may be an important alternative to breast-conserving surgery. Breast surgeons should pay much attention to not only perioperative complications and local control but also careful determination of RFA indication for breast cancer under the careful consideration of radiation-induced breast deformity and aging-related breast changes.

## Conclusions

We experienced an extremely rare case of late-onset cellulitis associated with an RFA-nodule more than 20 years after treatment. Breast surgeons need to determine the indication of RFA for breast cancer, taking into account the effects of radiation and aging on the breast. Additionally, some reduction mammoplasty to the contralateral breast has the insurance coverage problem but can offer better cosmetic outcomes for the deterioration of RFA- and radiation-induced breast cosmesis.
